# Clinicopathologic and outcome features of superficial high-grade and deep low-grade squamous cell carcinomas of the penis

**DOI:** 10.1186/s40064-015-1035-2

**Published:** 2015-06-09

**Authors:** Alcides Chaux

**Affiliations:** Department of Scientific Research, Norte University, Gral. Santos e/25 de Mayo, Asunción, Paraguay; Centro para el Desarrollo de la Investigación Científica, Asunción, Paraguay

**Keywords:** Penile cancer, Squamous cell carcinoma, Histological grade, Prognostic factors, Outcome, Anatomical level

## Abstract

**Purpose:**

To report the clinicopathologic and outcome features of superficial high-grade and deep low-grade penile squamous cell carcinomas.

**Methods:**

From a retrospectively-collected series of patients with penile cancer we identified 41 cases corresponding to 12 superficial high-grade tumors and 29 deep low-grade tumors. As outcomes we evaluated inguinal lymph node status, presence of tumor relapse, final nodal status, and cancer-specific death. Follow-up ranged from 0.8 to 386.7 months (mean 152.5 months, median 157.3 months).

**Results:**

Clinicopathologic features were similar between superficial high-grade and deep low-grade tumors, except for a tendency (Fisher’s exact $P=0.057$) of the former to include tumors with a verruciform pattern of growth. A significantly higher proportion of inguinal lymph node metastasis was found in superficial high-grade tumors compared to deep low-grade tumors [4/5 (80%) vs. 1/5 (20%) respectively, Fisher’s exact $P=0.02$]. No significant differences were found regarding tumor relapse (Fisher’s exact $P=0.52$), final nodal status (Mantel-Cox’s $P=0.42$), or cancer-related death (Mantel-Cox’s $P=0.52$).

**Conclusions:**

Patients with superficial high-grade tumors had a significantly higher proportion of inguinal lymph node metastasis compared to patients with deep low-grade tumors. On this regard, prophylactic inguinal lymphadenectomy might be indicated in cases of superficial tumors with high-grade histology while in deeply invasive low-grade penile carcinomas a more conservative approach may be considered.

## Background

Pathologic features of the primary tumor affecting outcome of patients with penile cancer are multiple and include histological grade (Chaux et al. 2009, 2009; Velazquez et al. 2008), percentage of anaplastic cells (Slaton et al. 2001), anatomical level of infiltration (Chaux et al. 2009), depth of invasion (Dai et al. 2006; Emerson et al. 2001), tumor stage (Slaton et al. 2001; Dai et al. 2006; Guimarães et al. 2006), presence of vascular (Slaton et al. 2001; Emerson et al. 2001; Guimarães et al. 2006; Ficarra et al. 2005) and perineural (Chaux et al. 2009; Velazquez et al. 2008) invasion, histological subtype (Dai et al. 2006; Guimarães et al. 2009) and tumor front of invasion (Guimarães et al. 2006). Risk groups systems combine histological grade with tumor extension to estimate the likelihood of inguinal nodal involvement (Solsona et al. 2001, 2004; Hungerhuber et al. 2006; Ornellas et al. 2008). The combination of histological grade, anatomical level of invasion and presence of perineural invasion was found to be strongly related to nodal involvement and cancer-specific survival (Chaux et al. 2009). As usually seen with tumors elsewhere, in penile cancer depth of tumor invasion and histological grade are frequently and significantly associated (Guimarães et al. 2009). However, we have occasionally found superficial tumors depicting a high histological grade and deeply infiltrating malignant neoplasms showing a low-grade histology. The purpose of this study was to evaluate the clinicopathologic and outcome features of patients with such “paradoxical” tumors.

**Table 1:** Clinicopathologic and outcome features of superficial high-grade and deep low-grade tumors

	Superficial high-grade	Deep low-grade	P value
No. cases (%)	12 (29%)	29 (71%)	
Clinical features			
Patients’s age in years, median (IQR)	52.5 (12.5)	56 (19)	0.11
Tumor size in cm, median (IQR)	4.5 (4.125)	5 (2.125)	0.95
Anatomical location (%)			0.89
Glans alone	7/26 (27%)	19/26 (73%)	
Glans + coronal sulcus	2/6 (33%)	4/6 (67%)	
Glans + coronal sulcus + forsekin	3/9 (33%)	6/9 (67%)	
Pathologic features			
Histologic subtype (%)			0.057
Usual SCC	11/24 (46%)	13/24 (54%)	
Verrucous carcinoma	0/7 (0%)	7/7 (100%)	
Papillary carcinoma	0/3 (0%)	3/3 (100%)	
Warty carcinoma	1/3 (33%)	2/3 (67%)	
Mixed carcinoma	0/4 (0%)	4/4 (100%)	
Urethral invasion (%)			0.7
Positive	3/15 (20%)	12/15 (80%)	
Negative	6/19 (32%)	13/19 (68%)	
Perineural invasion (%)			1
Positive	2/6 (33%)	4/6 (67%)	
Negative	10/34 (29%)	24/34 (71%)	
Vascular invasion (%)			0.57
Positive	2/4 (50%)	2/4 (50%)	
Negative	10/36 (28%)	26/36 (72%)	
Outcome features			
Inguinal lymph node metastasis (%)			0.02
Positive	4/5 (80%)	1/5 (20%)	
Negative	8/36 (22%)	28/36 (78%)	
Tumor relapse (%)			0.52
Local, regional or systemic relapse	1/2 (50%)	1/2 (50%)	
No tumor relapse	11/38 (29%)	27/38 (71%)	
Final nodal status (%)			0.2
Positive	4/8 (50%)	4/8 (50%)	
Negative	8/33 (24%)	25/33 (76%)	
Death by disseminated cancer (%)			1
Positive	0/1 (0%)	1/1 (100%)	
Negative	12/40 (30%)	28/40 (70%)	

P values were estimated using the Fisher’s exact test for categorical data and the Kruskal–Wallis rank sum test for grouped numerical data.

*IQR* interquartile range,* SCC* squamous cell carcinoma.

## Methods

### Cohort of patients

Patients were selected from a previously published series of 333 patients with invasive penile squamous cell carcinoma (Guimarães et al. 2009). This dataset is publicly available at http://dx.doi.org/10.6084/m9.figshare.1290997.

### Classification of cases

The dataset was searched for tumors fulfilling the following criteria:

**Superficial high-grade tumors**: grade 3 tumors invading lamina propria or superficial corpus spongiosum (tumor thickness $\le$5 mm).

**Deep low-grade tumors**: grade 1 tumors invading deep corpus spongiosum (tumor thickness $\ge$10 mm) or corpus cavernosum, including tunica albuginea.

The cutoff points of 5 and 10 mm were selected based on previous studies (Velazquez et al. 2008). Tumors not fulfilling these criteria were excluded from the dataset. From the full dataset of 333 patients, 29 patients (8.7%) were lost at follow-up, and thus excluded. The final count of selected cases for data analysis was 41, which included 12 (29%) superficial high-grade tumors and 29 (71%) deep low-grade tumors.

### Follow-up

Patients were followed-up from 0.8 to 386.7 months (mean 152.5 months, median 157.3 months). Two endpoints were evaluated during follow-up:

1. **Tumor relapse**: tumor relapse included the development of local relapse (i.e., tumor on stump), regional relapse (i.e., metastases in regional lymph nodes), or systemic relapse (i.e., metastases in systemic lymph nodes, visceral metastases) during follow-up.
2. **Outcome**: the possible categories of outcome included alive without disease, alive with disease, died of other causes, and died of cancer.

### Final lymph nodes status

The final status of inguinal lymph nodes was established as follows:

1. **Positive status**: the final nodal status was considered positive if pathologically-proven nodal metastases were observed (in those cases with inguinal lymphadenectomy), if regional relapse appeared during follow-up, or if the outcome was unfavorable (i.e., alive with disease or death by cancer).
2. **Negative status**: the final nodal status was considered negative if nodal metastases were not observed (in those cases with inguinal lymphadenectomy), if no tumor relapse (beyond local relapse) appeared during follow-up, or if the outcome was favorable (i.e., alive without disease or death by other causes).

### Statistical analyses

Bivariate analyses were carried out using the Fisher’s exact test for contingency tables and the Kruskal–Wallis rank sum test for grouped numerical variables. Survival curves were generated using the Kaplan–Meier method and compared with the log-rank (Mantel-Cox) test. Unconditional logistic regression models were built to estimate odds ratios and 95% confidence intervals. In all cases a 2-tailed $P<0.05$ was required for statistical significance. Data were analyzed and plots were generated using R version 3.2.0 “Full of Ingredients” (R Core Team 2015). The dataset and R scripts used for data analysis, as well as additional results (including the full analysis of the dataset), are freely available at https://github.com/alcideschaux/Penis-Paradoxical.

**Table 2:** Odds ratios and hazard ratios for superficial high-grade vs. deep low-grade tumors by outcomes

Outcome	Logistic regression			Cox’s regression		
	OR	95% CI	P value	HR	95% CI	P value
Nodal metastasis	14	1.8, 295.7	0.026	6.13	0.68, 55.02	0.065
Tumor relapse	2.5	0.09, 65.89	0.54	2.04	0.13, 32.62	0.54
Final nodal status	3.1	0.61, 16.27	0.16	1.77	0.44, 7.14	0.43
Death by cancer	3.3e−08	NA, Inf	1	3.2e−09	0.00, Inf	0.41

Odds ratios were estimated using unconditional binary logistic regression. Hazard ratios were estimated using Cox’s proportional hazards regression. Results for death by cancer are not evaluable due to the small number of events and should be discarded.

*CI* confidence interval,* HR* hazard ratios,* Inf* infinite,* NA* not available,* OR* odds ratios.

## Results

Table 1 compares the clinicopathologic and outcome features of patients with superficial high-grade and deep low-grade tumors. None of the clinicopathologic features were significantly associated with the type of tumor, although deep low-grade tumors tended to exhibit a verruciform pattern of growth. Of the outcome features, only the presence of inguinal lymph node metastasis was significantly different between superficial high-grade and deep low-grade tumors, with a higher proportion of metastasis in the latter [4/5 (80%) cases] compared to the former [1/5 (20%) cases].

Table 2 shows the results of the logistic and Cox’s regression analysis for predicting outcomes based on the type of tumor. Patients with superficial high-grade tumors had an significantly increased odds ratios for inguinal lymph node metastasis compared to patients with deep low-grade tumors. Hazard ratios were also increased in patients with superficial high-grade tumors compared to patients with deep low-grade tumors, although the P value was slightly above the standard threshold. Risks were not significantly different for tumor relapse or final nodal status. Risk for cancer-related death was not evaluable due to the small number of events.

**Figure 1:**
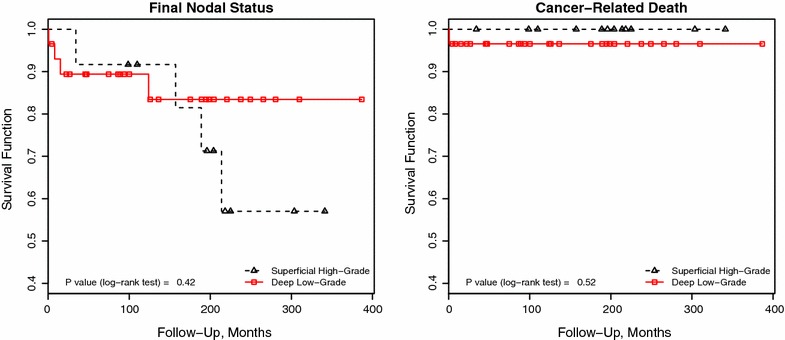
Kaplan–Meier survival curves for final nodal status and cancer-related death by type of tumor. No significant differences were found in the survival curves. Follow-up in months is depicted in the x-axes, while the y-axes depict survival functions. P values were estimated using the log-rank (Mantel-Cox) test.

Figure 1 shows the survival curves for final nodal status and cancer-related death by type of tumor. As seen, no significant differences were observed between patients with superficial high-grade and deep low-grade tumors in regards to the aforementioned outcomes. Individuals at risk for all survival curves are included as supplementary material in the online repository at https://github.com/alcideschaux/Penis-Paradoxical.

## Discussion

In this study we analyzed the clinicopathologic and outcome features of patients with superficial high-grade and deep low-grade squamous cell carcinomas of the penis. We found no significant differences in the clinicopathologic features, except for some tendency of low-grade tumors to exhibit a verruciform pattern of growth. Regarding outcome, superficial high-grade tumors showed a higher proportion of inguinal lymph node metastasis compare to deep low-grade tumors, suggesting that histological grade is more influential on prognosis than depth of invasion in this particular setting. Nevertheless, the type of tumor had limited usefulness in predicting nodal disease (i.e., final nodal status) or cancer-related death, indicating that other factors should be taken into account for predicting long-term outcome. Our results suggest that patients with superficial high-grade tumors may benefit from a more aggressive approach (v.g., prophylactic inguinal lymphadenectomy), in spite of their lower pT stage. Conversely, patients with deep low-grade tumors could be suitable candidates for an active surveillance program instead of a more aggressive approach, despite their higher pT stage.

Our results are in agreement with a previous study evaluating penile tumors invading 5–10 mm, in which histological grade had more influence on prognosis than depth of tumor invasion (Velazquez et al. 2008). Given its importance and clinical implications histological grading should be carried out using uniform and comparable criteria, moreover considering that a significant inter-observer variability has been reported for histological grading in penile carcinomas (Naumann et al. 2009). For patients included in this study, histological grading was carried out using strict (and previously validated) morphologic criteria (Chaux et al. 2009). This approach may reduce inter-observer variability, although further studies are required to evaluate the external validity and reproducibility of such criteria.

In addition, to consider only the T stage of the penile tumor to define the type and extension of primary treatment could be misleading. Some tumor variants, such as basaloid, sarcomatoid and high-grade usual carcinomas are intrinsically aggressive, regardless of the anatomical level of infiltration (Chaux et al. 2009; Velazquez et al. 2005; Guimarães et al. 2009). Other tumor variants, such as carcinoma cuniculatum, could invade deep erectile tissues and nevertheless be associated with good prognosis (Barreto et al. 2007). Moreover, the current TNM system for penile cancer considers invasion of corpus spongiosum or corpus cavernosum as a single pT2 category (Hakenberg et al. 2015). Previous studies have found that the metastatic rate of tumors invading corpus cavernosum is higher than those tumors limited to corpus spongiosum (Chaux et al. 2009). Furthermore, splitting the pT2 stage in two categories depending on invasion of corpus spongiosum vs. corpus cavernosum has proven useful in increasing the accuracy of the TNM system for predicting outcome (Leijte et al. 2008). It does not seem advisable to rely solely on the pT stage to plan optimal therapeutic management. An anatomical level-based approach, combined or not with measurement of tumor thickness, has been evaluated and found useful (Chaux et al. 2009; Velazquez et al. 2008). Information on histological grade should be included in pathologic reports and anatomical level of infiltration weighted with this grading in order to plan optimal management of patients with penile cancer.

We are aware that the classification we used (i.e., superficial high-grade vs. deep low-grade tumors) might seen incorrect taking into account risk groups systems such as the one proposed by the European Association of Urology (EAU). In the EAU system, both superficial high-grade and deep-low grade tumors would be classified as intermediate risk tumors (Solsona et al. 2004). Thus, both types of tumor would be managed with either dynamic sentinel lymph node biopsy or inguinal modified lymphadenectomy. However, in a recent study we have found that the EAU system shows a very low accuracy for predicting inguinal lymph node metastasis (Chaux 2015), and thus it might not be a reliable tool for therapeutic planning. These findings are consistent with other previous studies (Novara et al. 2008).

As mentioned before, tumors with a verruciform pattern of growth predominated in the deep low-grade category. Tumors with verrucous features includes verrucous carcinoma, papillary, and warty carcinomas (Chaux et al. 2010; Chaux and Cubilla 2012). Verrucous carcinomas were characterized by prominent acanthosis, inconspicuous fibrovascular cores, hyperkeratosis and a broad pushing base. It is noteworthy that, although verrucous carcinomas are considered as non-invasive tumors in the latest TNM classification system (Hakenberg et al. 2015), they can affect deep erectile tissues, as shown in this study. Low-grade warty and papillary carcinomas were very similar in their pattern of growth but papillae were more irregular and architecturally complex in papillary carcinomas and koilocytosis were easily found in warty carcinomas.

The superficial high-grade category included high-grade usual and warty carcinomas. High-grade usual SCC was characterized by nuclear pleomorphism, irregular nuclear membrane, coarse chromatin, prominent nucleoli and high mitotic rate. The other subtype found in the superficial high-grade category was high-grade warty carcinoma, a tumor depicting all the typical aforementioned features of a warty carcinoma (papillae with prominent fibrovascular cores, conspicuous koilocytosis, and jagged tumor base) but showing areas of anaplastic cells, usually at the tumor front of invasion.

At least two limitations must be acknowledged. First, our study is based on a retrospectively-collected series of cases. As such, clinical features were gathered form clinical reports and pathologic features were evaluated only on available microscopic slides. This might have had an impact in the quality of the data that is hard to estimate. Second, our population sample is relatively small, thus our study might be underpowered to detect the real impact of these tumor types on prognosis. This second limitation is bound to the relatively infrequent combination of low-grade and deep infiltration on one hand, and of high-grade and superficial infiltration on the other hand. Clearly, similar studies (preferably with prospectively-collected data) and on larger datasets of patients with penile cancer are required to determine if the differences and similarities we found are constant features of these type of tumors. Notwithstanding these limitations, our study suggests that patients with superficial high-grade and deep low-grade tumors might benefit from a different therapeutic management than the approach used in more usual clinical settings.

## Conclusions

In conclusion, whereas in the majority of penile carcinomas higher grade and deeper tumor invasion are significantly associated, there are cases in which a high-grade tumor may invade only superficially and low-grade tumors can affect deep erectile tissues. Tumors with such features were identified in about 8% of all cases in a large dataset of patients with penile squamous cell carcinomas. We found that superficial high-grade tumors had a significantly higher proportion of inguinal lymph node metastasis compared to deep low-grade tumors. On this regard, prophylactic inguinal lymphadenectomy might be indicated in cases of superficial tumors with high-grade histology while in deeply invasive low-grade penile carcinomas a more conservative approach could be considered.
